# Implement social prescribing successfully towards embedding: what works, for whom and in which context? A rapid realist review

**DOI:** 10.1186/s12889-024-18688-3

**Published:** 2024-07-09

**Authors:** C. Bos, E. de Weger, I. Wildeman, N. Pannebakker, P. F. Kemper

**Affiliations:** 1 National Institute for Public Health and the Environment (RIVM), Centre for Public Health, Care and Society, Department of health and Care Nationally, P.O. Box 1, Bilthoven, 3720 BA The Netherlands; 2 Vrije universiteit Amsterdam, Athena Instituut, de Boelelaan 1085, Amsterdam, 1081 HV The Netherlands; 3 TNO child health, Sylviusweg 71, Leiden, 2333 BE The Netherlands; 4 Research group Innovation in Preventive Healthcare, HU University of Applied Sciences Utrecht, Heidelberglaan 7, Utrecht , 3584 CS The Netherlands

**Keywords:** Social prescribing, Implementation, Embedding, Rapid realist review, Integrated care, person-centred care, Addressing wider health needs

## Abstract

**Background:**

Some clients who access healthcare services experience problems due to the wider determinants of health which cannot be addressed (solely) by the medical sector. Social Prescribing (SP) addresses clients ’ wider health needs and is based on linkworkers who support primary care clients in accessing social, community and voluntary care services that support their needs. Previous literature has provided valuable insights about what works (or not) in an early stage of implementing SP. However, there is limited insight into what works for the implementation of SP towards embedding. This study provides guiding principles by which SP can be successfully implemented towards the embedding stage and identifies which contextual factors and mechanisms influence these guiding principles.

**Methods:**

A Rapid Realist Review was conducted to examine what works, for whom, why, and in which contexts. A local Dutch reference panel consisting of health and care organisations helped to inform the research questions. Additionally, a workshop was held with the panel, to discuss what the international insights mean for their local contexts. This input helped to further refine the literature review’s findings.

**Results:**

Five guiding principles were identified for successful implementation of SP at the embedding stage: • Create awareness for addressing the wider determinants of health and the role SP services can play; • Ensure health and care professionals build trusting relationships with all involved stakeholders to create a cyclical referral process; • Invest in linkworkers’ skills and capacity so that they can act as a bridge between the sectors; • Ensure clients receive appropriate support to improve their self-reliance and increase their community participation; • Invest in the aligning of structures, processes and resources between involved sectors to support the use of SP services.

**Conclusion:**

To embed SP, structural changes on a system level as well as cultural changes are needed. This will require a shift in attitude amongst health and care professionals as well as clients towards the use, role and benefit of SP services in addressing the wider determinants of health. It will also require policymakers and researchers to involve communities and include their perspectives.

**Supplementary Information:**

The online version contains supplementary material available at 10.1186/s12889-024-18688-3.

## Background

Health systems worldwide are faced with clients’ increasingly complex health and care needs, growing health inequalities and increasing healthcare costs. These challenges have long underpinned the need to provide better quality and more person-centered care [1, 2]. People who access health and care services frequently experience problems due to the wider determinants of health which cannot be addressed (solely) by the medical sector [3]; e.g. clients experiencing psychosocial problems due to increasing levels of loneliness, uncertainty about finances, housing and unemployment [4–6]. Many of these clients receive inappropriate care because their non-medical needs are solely treated in the medical sector [7]. This may lead to overuse and undesirable costs [8, 9]. As such, there is an increasing awareness that identifying clients’ non-medical needs is crucial to tackling such problems [10]. To address clients’ wider, non-medical needs, more integrated and person-centered care is necessary. Health and care organisations (e.g. health, social care and voluntary sector providers, insurance companies, municipalities) are collaborating to implement new and more holistic models of care, such as Social Prescribing (SP).

SP is a relatively new approach which aims to address clients’ non-medical needs and is based on linkworkers who support primary care clients in accessing social care, community and voluntary services for their wider health and wellbeing needs. This approach aims to create a bridge between the medical sector and other sectors like the social, community and voluntary sector. SP includes direct signposting by general practitioners (GPs) to community and statutory services or to a linkworker for more intensive coaching interventions and then referred to the community [11]. There are a range of different models for SP. These vary in referral pathways, target groups (e.g. clients with psychosocial problems, complex multi-problems, chronic illness), and services and activities; (e.g. gym referrals, community classes, housing advice, gardening clubs, green health interventions) [12, 13]. Most SP models have linkworkers within the community [14, 15]. Linkworkers address clients’ wider health needs and, together with the client, decide which services are most appropriate. For example, linkworkers may support clients through coaching (e.g. to empower clients’ self-reliance), find an appropriate activity for clients to join, or services which can help solve their (non-medical) problems. Several studies suggest that clients who use SP services experience improvement in their well-being, their self-esteem, and self-reliance through the support of networks [16–18]. However, some literature indicates that such improvements are not a guarantee for all clients. Clients in more vulnerable circumstances (e.g. with severe mental health, financial problems, or housing issues) may find it difficult to attend SP services [19, 20]. Literature from the citizen involvement field suggests that vulnerable, ‘hard-to-reach’ clients face more structural and accessibility barriers, such as power imbalance, lack safe and trusting environment or not supported (e.g. financial) enough to be involved [21–25]. Such structural barriers and accessibility issues, if not addressed in the implementation or embedding phases, have the potential to increase health disparities when not properly designed [26, 27].

Previous literature has provided valuable insights about what works or not in the early implementation stages. However, insights and an overview of what works for the implementation of SP towards embedding is missing. An implementation process consists of different stages (i.e. orientation, insight, acceptation, change and embedding) [28] and should be seen as iterative rather than linear [29, 30]. This research focuses on the stages after pilot/orientation towards embedding. According to the normalization theory, embedding means that SP becomes so embedded into routine practices that it ‘disappears’ from view (i.e., it is normalized) [31, 32]. Insights in the early stages of implementation (i.e. as pilots) are, for instance, highlight the need for training about the meaning of SP for GPs, the importance of connecting GPs with linkworkers and the need for health and care professionals to learn and collaborate on behalf of SP [4, 33, 34]. However, SP services are being increasingly implemented worldwide and some health and care organizations are ready to move beyond the pilot phase and are looking for guidance to further implement SP towards embedding. For instance, in the Netherlands there are several models of SP that have been piloted which means organisations are now searching for how to further implement SP towards embedding in their own local contexts. Insights from other studies are helpful to reflect on what is needed throughout the implementation process towards embedding after SP models were launched as a pilot. However, while earlier literature studies provided valuable insights, these are mainly focused on pilots testing how the SP interventions work or are focused on the use of SP by one specific profession [4, 14, 35]. They do not give a complete, system-wide overview of what is needed to further implement SP to make it part of the daily practice of health and care professionals (embedding), and what works, why and in which contexts of social prescribing. To start providing such an overview, this rapid realist review (RRR) set out to develop guiding principles to successfully implement SP towards embedding. The principles are based on international literature about social prescribing and is a substantiation of which Social Prescribing interventions work, for whom, how, to what extent and in which contexts. The principles are useful for policymakers and the health and care professionals to explore when struggling with the implementation and embedding of social prescribing in their own context. The review addressed the following research questions:

1. What are the guiding principles by which social prescribing can be successfully implemented towards embedding?
2. What are the underlying contextual factors and mechanisms influencing these guiding principles?

## Methods

### Setting

This study is part of a larger action research study ‘wellbeing on prescription’ (a social prescribing model) from the Dutch National Institute for Public Health and the Environment (RIVM), in collaboration with the Dutch organization for Applied Scientific Research (TNO). This study aims to understand what is needed to further implement wellbeing on prescription towards embedding. The first phase of this study was this RRR to gain insights into the international literature and lessons learned. The second phase of this study is a participatory action study to follow different health and care organisations from four different regions to gain insight into the enablers and barriers during the implementation process to continue with embedding ‘wellbeing on prescription’ and which enablers should be considered. This paper focuses on the first phase of the study, the RRR.

### Research design

This study applied the rapid realist review (RRR) approach. The realist synthesis approach is an approach designed for analyzing complex programs of interventions [36]. It is used to gain a deep understanding of ‘what works for whom, in which context and with which outcomes’, based on the argument that the interaction between the contextual factors and mechanism has impact on the intervention outcomes (Pawson and Tilly, 1997). In the Realist approach the relationships between the context (C), the mechanism (M) and the outcome (O) are identified through context-mechanism-outcome configurations. For this study, configurations helped explain why implementing a social prescribing program is successful in context A, but not in context B [37, 38]. The aim of the RRR is the same as a traditional Realist Review, but it is a time-responsive method allowing the generation of findings to inform policy and involved stakeholders [39, 40]. This review followed several steps based on examples of Saul and Stolee and Willis, which are related to: developing & refining research questions, searching & retrieving information, screening & appraising information, synthesizing information and interpreting information [38, 40, 41].

The first step was to align the research questions with a reference panel. The reference panel for this study consisted of Dutch health and care organisations who were involved in the previously described participatory action research (see ‘setting’). These health and care organisations helped to inform the research questions. Because there are such wide ranging definitions and interpretations of SP, an important first step before the search process was to agree on one clear definition of the approach which the authors could then apply throughout each stage of the review. Based on a preliminary search of the literature and early consultations with the panel, the authors chose the following definition of social prescribing:

*‘a means of enabling general practitioners and other frontline healthcare professionals to refer clients to a link worker – to provide them with a (face-to-face or digital) conversation. During this conversation clients can learn about the possibilities of services or activities provided by the social, voluntary and community sector, so clients with social, emotional or practical needs are empowered to find solutions which will improve their health and well-being’ (based on SPN, 2016)* [42].

### Search process

**Fig. 1:**
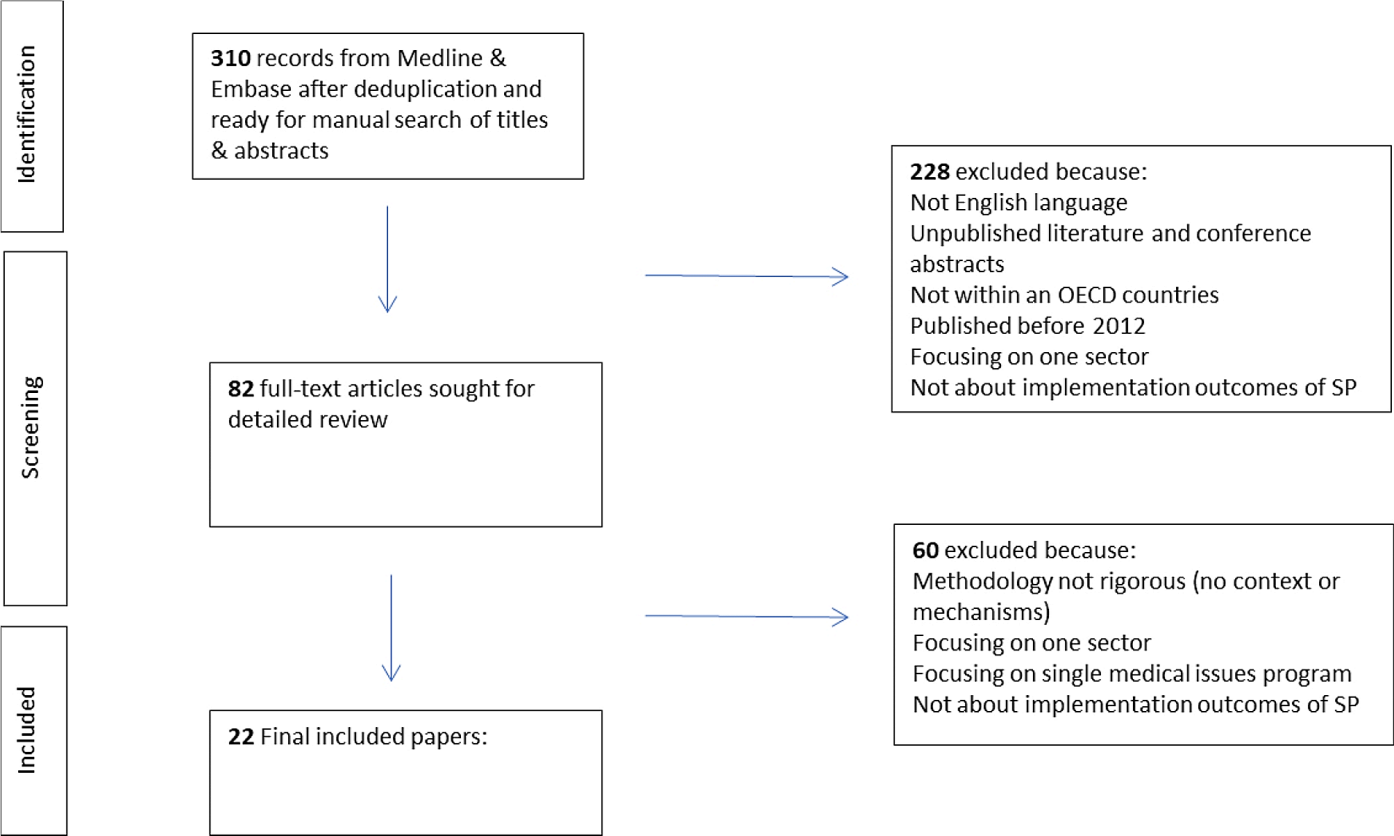
Prisma flowchart of document inclusion and exclusion process

In consultation with the library scientist at the RIVM, the review search terms and search strings were (See appendix 1) applied in the electronic database Medline and Embase. The search terms were based on the research of Mesman [43]. After the removal of duplicates, the search resulted in 310 potentially relevant papers (see Fig. 1). The inclusion and exclusion criteria were developed by the researchers (Fig. 1). The researchers (ChB and EdW) screened the papers in two stages. During the first stage the two researchers applied the criteria to the titles and abstracts of the included papers. The two researchers screened each of the papers and then cross-checked to discuss these papers. After the first title and abstract screening stage, 82 papers were selected to continue to the second full text screening stage (see Fig. 1). During the second screening stage, the two researchers assessed the full text of those 82 selected papers with the established criteria. Both screened the same 10 papers and then checked for inter coder agreement/ cross-checked the coding. After this each reviewer screened the rest of the papers and then cross-checked to discuss these papers. The Mixed Methods Appraisal Tool (MMAT) was used for methodological rigour and relevance [44]. Finally a total of 22 papers were included in literature review (Aughterson, 2020; Bertotti, 2018; Calderón-Larrañaga 2022; Carnes, 2017; Calderón-Larrañaga, 2021; Chang, 2021; Costa, 2021; Elliott, 2022; Fixsen, 2020; Gibson, 2021; Hazeldine, 2021; Holding, 2020; Islam, 2020; Khan, 2021; McHals, 2020; Pescheny, 2018; Pescheny, 2018, Rhodes, 2021; Scott, 2021; Thomas, 2021; Tierney, 2020; Wood, 2021).

### Analysis

Data extraction was conducted on the final set of 22 selected papers using the computer software programme MaXQDa. A coding tree was made based on existing literature, experts and professional opinion. The coding tree was used to analyse the intervention logic, strategy, lessons, professionals and clients’ needs, experiences and perceptions, interventions aspects and resources and determinants of implementation towards embedding. For transparency and to ensure consistency in the analysis of the realist concepts, the authors specified the definitions of the context, mechanisms and outcomes that were used in this study with a focus on the implementation of social prescribing towards embedding related definitions of important realist concepts (see Table 1). Two researchers (ChB and EdW) formed Context- Mechanism-Outcome (CMO) configurations within each of the papers. Each researcher formed CMOs in half of the papers and cross-checked the other half of the papers. The agreed upon CMOs were thematically clustered based on the combination of mechanisms and outcomes into overarching themes. ChB made a first draft of guiding principles based on these overarching themes. This draft was checked by EdW. These initial guiding principles were tested and validated with the Dutch local reference panel within a workshop.

**Table 1:** Definitions of the realist concepts

The following definitions of the realist concept were applied:	
Context	Pertains to the ‘backdrop’ of program, which can be understood as any condition that triggers or modifies the mechanism [37]. In this study examples of contextual conditions are pre-existing cultural norms or collaboration between different sectors or previous experiences with SP services.
Mechanism	The concept mechanism exist of two components resources and reasoning. The resources referring implemented activities, strategies and interventions and the reasoning is how people cognitive, emotional or behavioral responses to a resources [37]. In this study an example of mechanism are when healthcare professionals present SP services a solution to the problems of clients the reaction, it can demotivate some client when expected referred to the medical sector instead of the community.
Outcome	Refers to intended and unexpected program outcomes [37]. Examples of this study are implementation outcomes of SP, such as clients can dropping out and not engaging further with SP when the expected the medical solution.

## Results

All of the included 22 papers about Social prescribing have similar starting points, namely clients’ (wider) health needs, but the choice of focus regarding health problems and target groups varied. Examples of SP services are described in Table 2. For example, most of the articles focused on the activation of clients through short-term conversations with the linkworker combined with participation and connection to social and health activities. This is especially the case for clients with mild- psychosocial problems (e.g. loneliness) or clients with chronic health conditions who want to live healthier lives or are looking for meaningfulness [3, 4, 33, 45–49]. Other articles focused on providing clients’ longer-term problem-solving support through multiple sessions with the linkworker combined with the use of services. This is especially the case for clients who experience housing, financial, unemployment or serious mental health problems [14, 35, 47]. Finally, some articles focus on SP with a combination of activation and longer term problem-solving support for clients. This is especially the case for clients with multiple and complex problems. Such clients need help with solving their problems and reconnecting with their community, e.g. by joining an activity. Studies that focus on both were situated in high deprivation areas [13, 50].

**Table 2:** Overview of examples of SP services

Paper	Focus SP / target group	Involved stakeholders, sector	Activities
Aughterson, 2020	Clients with mental health problems	GPs, Voluntary and Community sector	Art-groups, peer support, walking clubs, community garden
Bertotti, 2018	Mild-mental health problems	GPs, linkworkers, community organisations	One-on-one sessions with linkworkers for coaching, motivation and listening. Social interaction between client and group of clients in running community initiatives. Social Interaction in other community initiatives
Carnes, 2017	Frequent flyers with depression, anxiety or social isolation. Clients who do not have acute crisis, uncontrolled addictions or uncontrolled mental health problems	Linkworkers, GP surgeries, third sector (not-for-profit) and community organisations	Maximum of 6 session with the linkworker Volunteers offer clients support as long as needed Exercise classes Cookery lunch clubs Library visits Church organised
Fixsen, 2020	Clients with low levels of depression, anxiety, loneliness, isolation, clients with chronic disease management issues, frequent flyers with perhaps non confirmed diagnosis but have stress, financial or housing problems	GPs practices, linkworkers, Voluntary, community sector	Sessions with linkworkers Activities for personal health goals (gym and exercise activities)
Gibson, 2021	Client aged 40–74 with different physical and mental health problems, such as depression, anxiety combined with diabetes. Financial problems Clients join SP for activation and motivation	Primary care, community and voluntary	Sessions with the linkworker for action plan and work on their goals Gardening, gym, social groups, walking groups, foodbank visits and financial help
Hazeldine, 2021	Serious mental health problems	Voluntary sectors organisations, GPs practices and linkworkers	Coaching sessions with linkworkers
Holding, 2020	Complex problems of loneliness (through physical problems), divers of mental health problems	Linkworkers and volunteers	Sessions with linkworkers to set goals which are monitored via phone calls and joint meetings with volunteers Matched with volunteers for a one-on-one relationship who signposts and accompanies clients to local community activities Assisting with benefits claims, legal issues and housing problems Join scrabble groups, reading groups, walk and talk groups and cycling groups
Khan, 2021	Clients 18+, unemployment or having a disability	Primary care, community organisations	Art and exercise, women and men groups, lunch clubs, walking groups, mental health support groups, art or crafts activities
Pescheny, 2018	Depression, anxiety, unemployment, loneliness, recovery of physical activities, weight loss, housing problems	General practices, linkworkers	Job center support, physical activity, social activity, mental health services, support groups, physical activity, community groups, massage therapy, wight management, housing and legal advice
Wood, 2021	Adults 18 + Depression Multi-morbid chronic physical health conditions Complex Social Issue Isolation	Community non clinical organisations with staff members specialize in different areas in a high socioeconomic deprivation area	Support with housing, benefits and enjoy supported networks

A total of five guiding principles for implementation of SP towards embedding were identified through the literature, and validated by the panel’s input. Appendix 2 gives an overview of the CMOs underpinning the principles (full list of CMOs available upon request). The following sections first describe each principle using the evidence from the literature review. It is important to note that the guiding principles do not stand alone, but are seen as an interconnected set to improve the implementation of SP towards embedding. After the principles, the panel’s reflections will be summarized. The panel’s reflections did not change the wording of the principles and instead triangulated and enriched the literature findings. Table 3 summarizes the key lessons and practical tools applied in interventions, enabling contexts and mechanism underpinning the principles that policymakers and health and care organisations can build on successfully implement SP towards embedding. It is worth noting that constraining contexts and mechanisms are largely two sides of the same coin [51].

### Guiding principle 1: Create awareness for addressing wider determinants of health and the role social prescribing services can play

For the successful implementation of SP towards embedding several articles discussed the need for creating awareness of the importance of addressing clients’ problems based on the wider determinants of health and for the fact that SP services are a valid treatment option. A shift in attitude of all involved stakeholders (the whole health and landscape including clients themselves) towards the use of SP services is needed [14, 33, 47, 48, 52–54]. Health and care professionals nor clients are familiar with the need to investigate problems related to the wider determinants of health underlying health complaints [46],This is especially important in deprived areas because there are more clients who experience multiple and complex problems affecting different areas of life [13, 14, 35, 47, 50]. Thus professionals and clients should be encouraged to think beyond the medical domain. When both are aware of addressing the (wider) health needs and recognize the value of operating with other sectors outside the medical domain, they are more likely to use SP services [33].

Several articles describe that the use of SP services by healthcare professionals and clients is uncommon [48, 52, 54]. For example, some professionals are unfamiliar with what SP services can offer and do not always recognize the value (e.g. joint activities in the local community as a therapeutic resource). One of the articles shows that health and care professionals are mostly driven by high quality of care for clients (mechanism). Thus, when aiming to recognize the value of SP services in a context where general practitioners are asked to refer clients to a linkworker, or to join an activity in the community (*context*), they get regular feedback about how the client was getting after their initial referral and have some positive experiences with these referrals (*mechanism*). This positive experience, related to the quality of care caused the general practitioners to use SP services more and were also more engaged (*outcome*) [4]. Reversely, when GPs require formal evidence regarding the effective of SP services on client outcomes (*context*) and there is too much focus on gathering formal evidence which may create less attention for actually addressing clients’ (wider) needs (*mechanism*), because clients need to fill continuous questionnaires which results in research getting in the way of offering clients appropriate support based on the (wider) needs (*outcome*) [4]. Such increased awareness will enable health and care professionals to better explain what SP services entail and how referrals to other sectors can help address clients’ wider needs. For example Pescheny, et all (2018) describe one example of when health and care professionals are not fully aware of what SP services entail they provide clients with inconsistent and incomplete information which creates confusion and false expectations about what SP services have to offer [53].

### Guiding principle 2: Ensure health and care professionals build trusting relationships with all involved stakeholders to create a cyclical referral process

Successful implementation of social prescribing towards embedding requires building trusting relationships with all involved stakeholders (e.g. GPs, linkworkers, volunteers, clients and communities) to create a cyclical referral process to help clients with different (wider) needs and to react when updating and revising plans based on clients’ changing needs, so clients can move back and forth across settings and sectors according to their (changing) needs [4, 12, 55].

Creating shared understanding about expectations (e.g. task descriptions and roles) regarding all involved health and care professionals is required because this creates clarity about the collaboration and referral processes across sectors (including the community and voluntary sectors). In addition, setting up new signposting and referral processes, either through setting up entirely new pathways or by integrating within existing pathways (*context*) requires residents and organisations from the local community to build relationships and work together with health and care professionals to improve shared understanding about SP and increase confidence about new activities or interventions for clients (mechanism). This makes it more likely to get new and more referrals to new (SP) activities or interventions (*outcome*) [12]. An key enabler is to create shared leadership between different sectors to create a cross organizational collaboration [34]. Furthermore, an enabler is to create a learning environment with all involved stakeholders to learn and interact with each other [34].

Furthermore, trusting relationship with clients is very important. When clients trust their health and care professional(s), on the one hand they feel safe enough to share their problems about their daily lives so all problems of the wider determinants of health can be addressed and on the other hand they are more willing to participate in a recommended community services [33, 56]. An enabler which contributes to clients’ trust in SP services is that SP services can maintain continuity in local services, particularly a low turnover in health and care professionals. This way the professionals have an established relationship with each other and can collaborate better around clients’ needs [4]. For clients a key enabler is that the linkworker comes from the same local community. Such linkworkers can help build trust between SP services and local community, which in turn helps to engage clients with SP services and helps to align services to community needs [49]. Finally a key enabler to build trusting relationships is having enough time to spend with clients. This means taking enough time to have face to face connections between all those involved stakeholders and taking the time to address clients’ wider determinants of health [4, 15, 33, 46].

### Guiding principle 3: Invest in linkworkers’ skills and capacity so that they can act as a bridge between the sectors

SP linkworkers can play a key role as a bridge between the sectors to support clients and to and refer them to the services or activities most appropriate for their (wider) needs. To fulfill this role linkworkers must be able to [1] understand and acknowledge the challenges organisations from different sectors face to be able to connect these organisations to organize care and support around the (wider) needs of clients [2], to create an overview of the locally available social infrastructure (e.g. services and activities) and ensure that their knowledge of available services is always up-to-date [3], to provide support to all kinds of (vulnerable) clients and communities with complex needs by coaching to create behavioral change and by addressing their (wider) health needs to find an appropriate services or activity [33, 45, 48, 50, 54, 55, 57]. Several articles discussed the role of linkworkers in social prescribing initiatives [14, 33, 35, 45]. In these articles, linkworkers have different backgrounds (e.g. psychotherapy, psychology and coaching), but mostly linkworkers do not have (professional) specific backgrounds, or work as volunteer.

According to the literature multiple skills are required to able to act as a bridge between the sectors. An example of when linkworkers organize the care and support around the needs of clients described by Calderon– Larrangaga, et al. 2022. SP is largely dependent on the infrastructure of local communities including the availability of community activities and transport which varied across localities (context), so when there are no available services some linkworkers use innovative strategies by developing self-sustainable groups around the clients’ interest linkworkers which motivate clients to join these self-sustainable groups (mechanism), this makes it possible for clients to join an activity or meet clients with the same needs (outcome) [55].

Investment in specific skills (e.g. coaching, active listening, motivational techniques) and capacity (e.g. enough time) is important, to enable that linkworkers can receive and help a large variety of clients with different ages and problems. However, to deal with these varied groups of clients, referrers have to stay on top of inappropriate referrals due to a lack of immediately accessible alternatives (e.g. long waiting list for statutory services) to prevent the linkworker become overstretched [45, 56]. Furthermore, investment is important so that other stakeholders involved in SP services see linkworkers as competent professionals to whom clients can be referred. Seeing a linkworker as a competent professional strengthens other stakeholders’ confidence and belief in SP services and helps them to accept and support the role of linkworker [56]. For clients, especially with multiple and complex needs, it is important to have contact with one person (linkworker) who has a network with stakeholders from different sectors and know where to refer a client onto based om their (wider) needs. For example, Bertotti, et al. (2018) describe that when clients with multiple and complex needs are referred to a linkworker (context) and linkworkers are empathetic with good knowledge of social support infrastructure available locally, it gives clients a sense of agency and control over their time with non-imposing support (mechanism). This has a beneficial impact on the client and especially for clients with multiple and complex needs (outcome) [33].

To fulfil the role as linkworker can be complex and demanding so different ways of support should be offered. To support linkworkers’ skills-development it is important to offer education, training and courses [14, 33, 47, 55, 56]. Another enabler to support growth in their role is offering supervision or peer to peer support, so they can share experiences and difficulties related to their role [3, 35]. Finally, an enabler is offering support by management to meet the (wider) needs of clients (e.g. help with in house clearance) [50].

### Guiding principle 4: Ensure clients receive appropriate support to improve their self-reliance and increase their community participation

Providing clients appropriate support may increase the chance to improve their self-reliance and stimulate the use of SP services [50, 55, 58]. There are a lot of clients who regularly visit the healthcare sector where problems surrounding their wider health needs are not adequately addressed or solved. Especially in vulnerable neighborhoods clients often experience multiple problems in different areas of life (e.g. financial, housing, domestic problems or trauma’s from the past) and also a lack of connection to a social network. With such problems it is important that problems related to livelihood security are solved in combination with participation in activities the community. When problems related to livelihood security regarding their financial security (e.g. unemployment, debt) are solved, clients feel more confident to participate in the community and feel less dependent on healthcare professionals [55]. For example Gibson, et al. 2021 describe how linkworkers try to remove feelings of discomfort and unfamiliarity with new situations (context), but clients’ deeper feelings about the past remain. Because of these persistent feelings of uncertainty and discomfort linkworkers are not always enough to help clients feel prepared enough for new and unfamiliar fields of practices, (mechanism). This means often time another intervention is needed first before such clients can participate in a community activity (outcome) [13]. However, when SP services are aimed at addressing wider determinants of health (context) and clients experience a lack of self-perception, motivation and confidence it is seen by health and care professionals as a barrier for successful engagement and behavioral change and makes clients deemed too dependent on SP services (mechanism) which is thought as a threat to SP implementation and delivery (outcome) [55].

To stimulate self-reliance there are different ways to support clients. Health and care professionals can support clients by informing them well so clients can take the next step themselves and find their way in all different sectors. Some clients need a boost to join SP services or community activities. So when clients are referred to SP services (context) and linkworkers contact clients after receiving a referral and give emotional and practical support to overcome barriers that prevented them from engaging (mechanism) and prevents dropouts and enables people to push themselves harder than they would have by themselves and they were more likely to participate (outcome) [55]. Clients also feel supported when they meet other people in similar situations, as they can support each other in an informal manner and share their experiences. Furthermore, another key enabler to support clients is to give them funding or give them access to transport to join activities [14, 45].

### Guiding principle 5: Invest in aligning structures, processes and resources between involved sectors to support the use of SP services

To improve the referral processes to SP services investment in shared resources and structural finances for SP services are necessary to make involved sectors less fragmented and improve opportunities for collaboration and communication [14, 45, 57]. Currently, the medical sector has arguably more formalized governance structures compared to other sectors (e.g. social, community and voluntary) regarding (e.g. confidentiality, data storage, (data) infrastructures, ways of processing referrals, staff training requirements or structural finance). For example, Wood et al. 2021 describe that many SP services and activities take place in the voluntary sector and are isolated from the formalized processes and structures (context) the lack of professional status and standards of SP staff prevent them from using the same resources as the medical sector (mechanism), which leads health and care professionals to have relevant information about what they can or cannot share with them and so prevent clients from constantly having to retell their story (outcome) [14, 45, 50, 54, 57, 59]. An enabler is to create clear guidance, standards for SP services and professional standards for linkworkers to improve the alignment between involved sectors and support the use of SP services [48, 50]. Furthermore a key enabler is to invest in a clear line of accountability and governance between all involved stakeholders of SP services at all stages of the process [48, 50].

Moreover, to improve the referral process to the social, community and voluntary sectors which ensures that referrals to traditional services are not the first default option. For example Scott, et al. 2021 describe (context) that triage and referrals pathways are key determinants of SP in prehospital care as these help identify to which services a potential clients can be referred (context) the lack of an automated system to other sectors causes referrals to traditional services because it feels time consuming (mechanism), which lead to reduced referrals to SP services and highlight the potential need to redraw referral pathways to better include SP services (outcome) [54]. So a key enabler is an automated digital system between all involved sectors and making sure that all involved SP stakeholders have access. Furthermore, a key enabler is to improve the referral process with general practices who offer linkworkers an open environment and practices support by offering a suitable location (e.g. access to practices to speak with practice staff and access practice resources; information system, advertising in waiting rooms) [14].

It is important to formalize SP services and make structural funding possible for SP service, because due to a lack of investment (e.g. structural funding) SP services and community activities cannot continue [4, 49] The continuity of activities and services is important for the trust of clients as well as health and care professionals. For example Khan, et al. (2021) describe short-term funding and the corresponding closure of organizations and activities negatively impacting citizens trust to join local services [49]. In addition when community activities are short-lived, it makes it difficult for health and care professionals to keep up their knowledge about local services up to date [33].

**Table 3:** Summary of actions and enablers for a successful implementation towards embedding of SP

Guiding principles and main lessons			
**Action or intervention**	**enabling context factors**	**enabling mechanisms**	**Citations**
*Create awareness of addressing wider determinants of health and the role social prescribing services can play* • *Ensure there is some (formal) evidence about the effectiveness and improvement on clients outcomes so health and care professionals as well as clients are more engaged.* • *Ensure that referrals to SP services are as normal as a referral to the medical sector to create a positive attitude to health and care professionals as well as clients.*			
• Provide feedback for professionals about processes of clients after referral (e.g. in regular meetings or a short periodic report) • Provide professionals with formal evidence which demonstrate effectiveness of S • Explain as professionals, especially as GP, clients with confidence about SP services • Help clients to explain their needs to stimulate more ownership instead of tell them what the client need to do.	• Professionals are curious and have a positive attitude to learning with each other • Good relationships between all involved partners helps communication about health outcomes • Health and care professionals and clients are familiar with the concept of SP services	• Health and care professionals are more likely to use SP services when clients were seen to be benefiting because they are mostly driven to high quality of care for clients • Health and care professionals are motivated to screen on (wider) needs when clients’ needs are unmet with medical options • When clients’ needs are unmet in the medical sector professionals feel enjoyable to help them in a different way • The role of the linkworkers is for all first line professional clear so they know what to expect and refer clients with confident	• Aughterson & Baxter, et al. (2020) • Bertotti & Frostick, et al. (2018) • Calderón- -Larrañaga, et al. (2021) • Costa & Lopez, et al. (2021) • Fixsen & Seers, er al (2020) • Hazeldine & Gowan, et al. (2021) • Mofizul Islam, (2020) • Scott & Fidler, et al. (2020) • Penschany & Pappas, et al. (2018) • Wood, et al. (2021)
*Ensure health and care professionals build trusting relationships with all involved stakeholders to create a cyclical referral process.* • *Create a shared understanding between all involved stakeholders which makes referrals more easily.* • *Ensure clients trust their health and care professionals so they share their problems and are more willing to participate in recommended SP services.*			
• Create shared understanding about expectations regarding all involved stakeholders • Make a clear pathway with stakeholders who involved in SP • Set out a clear like of accountability between all involved stakeholders of SP services at all stage of the process • Create shared leadership between different sectors to create a cross organizational collaboration • Deploy a linkworker who comes from the same local community	• Permanent staff to work with to make real preexisting relationships with stakeholders and clients • Clarity between the stakeholders about the collaboration and referral processes across sectors • SP seen as a care network of comprising different actors • Face to face sessions with enough time for each consultation with the linkworkers • There is now suspicion and rivalry on the part of some colleagues within buy-in stakeholders or there are doubts about the skills of linkworkers	• Professionals have enough time to make face to face connections with other professionals and the local community to know each other well • Professionals have enough time to address the (wider) needs of clients and clients feel save enough to share sensibilities about their personal lives • Trusting relationship with the local community makes that professionals can motivate clients to engage with community services or linkworker • General practitioners are proponents and have trust towards SP and believe linkworkers and community organisations can play a role in addressing clients’ needs • Shared leadership between the different organizations empowered teams to establish their own cross-organization relationships	• Aughterson & Baxter, et al. (2020) • Bertotti & Frostick, et al. (2018) • Calderón- -Larrañaga, et al. (2021) • Chang & Hawkins, et al. (2021) • Fixsen & Seers, er al (2020) • Khan & Ward, et al. (2021) • McHale & Pearsons, et al. (2020) • Pescheny, et al. (2018) • Tierney & Wong, et al. (2020)
*Invest in linkworkers’ skills and capacity so that they can act as a bridge between the sectors* • *Ensure linkworkers are able to (1) connect different organisations, (2) create an overview of the local infrastructure, (3) provide support to (vulnerable) clients with (multiple) health needs.* • *Ensure linkworkers are supported enough in various ways to feel confident enough to fulfill these job*			
• Connect different organisations with each other to provide the right care for clients • Create an overview of the local infrastructure • Provide support to (vulnerable) clients with (multiple) health needs • Be a temporary holding place for clients who cannot access immediately an other professionals • Facilitate that linkworkers works in the same building as other front line professionals • Provide linkworkers with enough support through education, supervision and peer to peer support • Gives client a sense of agency and control over their time with non-imposing support	• Linkworkers have enough time to fulfill their job and make the bridge between the different sectors • Linkworkers have a mix of skills when they have sessions with clients who experiences multi-problems • Innovative linkworkers with highly developed skills and relationships both with external organisations and their service-users	• Link workers feel supported enough by management to work with a person centered approach • Link workers feel supported enough so they can full fill these demanding job with confident • Give clients an intensive coaching intervention to overcome barriers and change behavior before moving to the next step • Linkworkers demonstrated genuine passion and drive and used innovative strategies to develop activities that are missing in the community infrastructure	• Aughterson, et al. (2020) • Bertolli & Frostick, et al. (2018) • Calderón- -Larrañaga, et al. (2021) • Carnes, et al. 2017 • Fixsen, et al. (2020) • Hazeldine, et al. (2021) • Holding, et al. (2020) • Pescheny, 2018 • Islam, et al. (2020) • Rhodes, et al. (2020) • Scott, et al. (2021) • Thomas, et al. (2021) • Thierney, et al. 2020 • Wood, et al. (2021)
*Ensure clients receive appropriate support to improve their self-reliance and increase their community participation* • *Ensure that clients receive appropriate support in resolving multi-problem issues to feel less dependent on health and care professionals* • *Make sure clients feel adequately supported to participate in social, community and voluntary services and activities*			
• Involve the informal care (community and voluntary) more within SP • Provide clients with the right information so they know what to expect from SP services • Guide clients with emotional and practical support so they participate in the community • Offer clients peer-to-peer support to meet people with the same needs • Contact clients directly after receiving a referral	• Enough services/activities to offer so clients receive appropriate support based on their needs • Clients have an open attitude and are motivated to use SP services • Clients feel confident enough to join activities • Clients have access to resources like money, transport to join SP services	• Makes it possible for SP staff to accommodate for all the referrals regardless of their number • Enough space for professionals to be flexible to tailor support to clients’ needs so clients can receive the care they need and feel supported • Peer to peer support amongst clients created that clients can support each other in an informal manner	• Aughterson, et al. (2020) • Bertolli & Frostick, et al. (2018) • Calderón- -Larrañaga, et al. (2021) • Dayson, et al. 2020 • Gibson, et al. (2021) • Hazeldin, et al. 2021 • Holding, et al. (2020) • Khan, et al. (2021) • Penscheny, et al. (2018) • Wood et al. (2021)
*Invest in the aligning of structures, processes and resources between involved sectors to support the use of SP services* • *Ensure there are shared resources and systems available between the sectors to collaborate and communicate across a diverse group of stakeholders.* • *Ensure there is structural funding so SP services can be offered permanently*			
• Defund professionals with time to organize pre-conditions for the implementation SP be offering project management for all the coordination • Establish a collaborative multi-sector approach with a diverse group of stakeholders • Make sure there are shared resources and (digital) systems between sector (community and voluntary • Create clear guidance and standards between health and care professionals	• Sustainable SP services delivered across different organizations • Sustainable long-term funding for the delivery of community activities • SP staff are considered as professionals that can help clients with (wider) needs • SP organisations are no longer isolated from statutory services	• Other health and care professionals feel confident to refer clients to SP staff • Information is passed between organizations in a timely manner preferably with an shared IT system which makes communication with each other easier • General practices are an open environment for linkworkers by offering practical support (e.g. location, access to speak with practice staff and practice resources)	• Aughterson & Baxter, et al. (2020) • Bertotti & Frostick, et al. (2018) • Calderón- Larrañaga, et al. (2021) • McHals, et al. (2020) • Hazeldine, et al. 2021 • Holding, et al. (2020) • Islam, et al. (2020) • Khan, et al. (2021) • Scott, et al. 2021 • Thomas, et al. 2021 • Wood & Ohlsen, et al. (2021)

### Local reference panel reflections

The guiding principles were tested and validated with the Dutch local reference panel. The panel recognized the guiding principles within their own contexts. During the panel discussion there were many similarities, but also some refinements and additions to the guiding principles. An important point that panel members mentioned is especially clients who are still unfamiliar with the concept SP of services and are also unfamiliar with what organisations in the local community can offer. Clients were said to often have other expectations when they visit a GP with what they perceive to be physical/medical complains. In such cases, panel members felt it was important to highlight that another professional, besides the GP, can help clients further with problems about their (wider) health needs. In addition, the panel members mentioned the importance that of the linkworker works in the same building as the primary care professionals. This was thought to improve the referral process by enabling healthcare professionals to know each other and know who they are referring clients to. Also it prevented client drop-outs because when they have to go to other places there is a higher chance they are not going. Another important validation of the literature findings is that the panel recognized the three main tasks of linkworker to enabling them to act as a bridge between sectors.

A refinement to the literature findings is that apart from being aware of SP services and activities it is also important to connect more with the local and informal networks in the community and to maintain relationships with e.g. community-led initiatives. Another refinement is that it is important that clients feel they have ownership of their wider health needs, because SP is centered on the idea that it is their needs which are central to find appropriate activities or services. Some new and additional information based on the results that panel members mentioned is that clients have sometimes lost confidence in the community sector because they did not get the help they needed or that choices through the community sector can be made that do not meet the needs of the clients.

## Discussion

While aiming to provide insights for further implementation towards the embedding of social prescribing, results in this study showed that most experiences with SP are aligned with the middle stages (e.g. insights, acceptation and change) of the implementations process [28]. Embedding SP into routine practice remains a challenge. This is in line with findings in the wider integrated care literature which shows that most studies of implementation relate to action for implementation of integrated care. These on the target group and service delivery, but not to the system level [60, 61]. In line with the wider literature, the guiding principles also found that to properly implement SP towards embedding, changes on different layers, macro (system integration), meso (organisations, professional) and micro – level (citizens, clients), are needed [62]. For example, the guiding principle about investing in alignment of structures and processes, and the panel’s input, show that health and care professionals who work with SP still have difficulty with preconditions among others on system level to make SP part of their daily practice. This seems to indicate that structural embedding is possible after changes on the system level have been implemented (e.g. finances, data infrastructure).

In addition to structural changes on a system level cultural changes (e.g. different ways of working) are necessary for further implementation of SP towards embedding. Reflecting on the NPT [31, 32] organisations and health and care professionals are not ready for embedding when, for example, they still need reminders about the existence of SP and when there is a lack of shared understanding about the daily operation of SP. Also health and care professionals receive limited feedback and proof about the impact on clients outcomes. Highlights the benefits to clients outcomes may help change attitudes towards the use of SP, because health and care professionals are motivated to offer clients appropriate support. Clients and communities are not ready for embedding because they do not always know about the existence of SP services and do not always know other ways than access the healthcare system then GPs despite their non-medical needs. For example, culture changes (e.g. stimulate working together with volunteers and residents of the community), some preconditions (e.g. space to collaborate and get to know each other) work through all layers (system, organizational, professionals and clients) and depend on each other.

Despite SP being centred on referrals to social, voluntary and the community sector, this study highlights that there is much unknown about the role communities and community-led initiatives can play, or to collaborate more successfully with them [14, 33, 45]. The role of the community is important because this study show that unfamiliarity with the community may hinder the use of SP for health and care professionals as well as clients, and also as long as the community and voluntary sector services are underrepresented GPs will always be first point of contact for clients. Khan, et al. (2021) highlight the importance of the collaboration with people in the community because they have the local knowledge and insights that help support the delivery of SP to better meet the needs of clients [49]. Furthermore, it is important when designing or further implementing SP services towards the embedding stages, that the needs of more vulnerable and ‘hard-to-reach’ groups are considered. According to de Weger (2022) and Cyril (2015) it is important to make enough space, by reaching out to these groups on their own terms to share their experiences, ideas and needs to improve SP services [22, 24]. Without giving special consideration to the needs and priorities of such groups, SP services may end up actually increasing health inequalities as SP services [24, 25] Moreover, Stathi, et al. 2021 highlight the importance of the value of peer volunteers in community initiatives [63]. To build a real community approach the reference panel suggest, for example, not only networking with formal services and activities is important but also connecting with the local informal networks and maintaining relationships with them. De Weger (2022) suggest investing in cultural changes to help community-led initiatives to flourish in the roles they wish to take on themselves (e.g. extend their role and contribute more) [21].

### Strengths and limitations

A strength of this study is that the guiding principles are validated with health and care organisations that work with SP in practice. However, important to mention is that the reference panel only consisted of Dutch health and care organisations, which makes that international lessons are only tested and refined by the Dutch context. Despite the validation through the Dutch panel it is also useful for other countries because al guiding principles based on international studies were recognized. However, to be sure each country can use the guiding principles as well as all context factors and mechanism it can be used as starting point to test the meaning in their own context.

### Future research

The guiding principles and their underlying CMOs are created for successful implementation towards embedding of SP. Future research can focus on how these five guiding principles can affect various stages of the implementation process. Also future research can focus on the connection with the community and their role within SP which also includes the perspectives of the community. Finally, future research should focus more on which changes in the system could contribute to making SP part of the daily practice of organisations, professionals, people and communities.

## Conclusions

The study demonstrates which principles can be followed for a successful implementation towards embedding of social prescribing. Most experiences are still aligned with the early and middle stages of the implementation process. To embed SP, structural changes on a system level are needed as well as cultural changes towards the attitude of using SP. By highlighting the contextual factors and mechanisms which influences the implementation outcomes of SP, the five guiding principles can guide policymakers and health and care professionals for a successful implementation of SP. While these contextual factors and mechanisms focus on health and care professionals, future policymakers and researchers need to be encouraged to include the role of the community and their perspectives to be sure SP services meet the needs of clients who need it the most. This paper provides valuable insights to embed and normalize SP services, which can bridge the gap between the medical and non-medical sectors. Ultimately this is needed to transform our health and care systems to become more person-centred and holistic.

### Electronic supplementary material

Below is the link to the electronic supplementary material.

Supplementary Material 1

Supplementary Material 2

## Data Availability

Full list of individual CMO configurations available upon request. Data request can be made by the authors.

## Abbreviations

SP: Social Prescribing
RIVM: National Institute for Public Health and the Environment (Dutch: Rijkstinstituut voor Volksgezondheid en Milieu)
TNO: Netherlands Organization for Applied Scientific Research
CMOs: Context-Mechanism-outcome configurations
MMAT: Mixed Methods Appraisal Tool
RRR: Rapid Realist Review
GPs: General Practitioners
