# Supplementary material for: Implement social prescribing successfully towards embedding: what works, for whom and in which context? A rapid realist review
Source: BMC Public Health. 2024 Jul 9;24:1836. doi: 10.1186/s12889-024-18688-3 (PMC11234751; doi:10.1186/s12889-024-18688-3)
Supplement: Supplementary file 1 — Supplementary Material 1 [file 12889_2024_18688_MOESM1_ESM.docx]

**Appendix 1: Search Strings**

| **“social prescrip*"[tiab] OR “social prescrib*"[tiab] OR "social refer*[tiab]" OR "well being program*" OR "wellbeing program*" OR "non medical refer*"[tiab] OR "community refer*" "non medical care*"[tiab] OR "referral to social services" [tiab]** **AND implem* OR facilitator* OR barrier* OR innov* OR evaluat* OR sustain* OR optimiz*** |
| --- |

#20 #18 NOT #19 35 🡪 Endnote WOR-3

#19 #18 AND 'Conference Abstract'/it 8

#18 #12 AND #16 AND ([dutch]/lim OR [english]/lim) 43

#17 #12 AND #16 43

#16 standard*:ti,ab OR component*:ti,ab OR determinant*:ti,ab 3,587,220

#15 #13 NOT #14 140 🡪 Endnote WOR-2

#14 #13 AND 'Conference Abstract'/it 26

#13 #7 AND #11 AND ([dutch]/lim OR [english]/lim) 166

#12 #7 AND #11 168

#11 implem*:ti,ab OR facilitat*:ti,ab OR barrier*:ti,ab OR innov*:ti,ab OR 7,857,149

evaluat*:ti,ab OR sustain*:ti,ab OR optimi*:ti,ab

#10 #8 NOT #9 306 🡪 Endnote WOR-1

#9 #8 AND 'Conference Abstract'/it 54

#8 (#1 OR #2 OR #3 OR #6) AND ([dutch]/lim OR [english]/lim) 360

#7 #1 OR #2 OR #3 OR #6 370

#6 #4 AND #5 32

#5 'psychological well-being'/exp/mj OR 'psycholog* well-being*':ti OR 7,501

'psycholog* wellbeing*':ti OR 'social needs'/exp/mj OR 'social need*':ti

#4 'health care utilization'/exp/mj OR 'health care utilizat*':ti 22,908

#3 'wellbeing program*':ti OR 'well-being program*':ti OR 'non medical refer*':ti OR 'non 143

medical care*':ti OR 'community refer*':ti OR (('referral*' NEAR/2 'social service*'):ti)

#2 'welzijn op recept*' 2

#1 'social prescribing'/exp OR 'social prescri*':ti 196

1 social-prescri$.ti. 47

2 (wellbeing-program$ or well-being-program$).ti. 24

3 exp *Well Being/ and ((wellbeing$ or well-being$) and program$).ti,ab. 3485

4 (exp *Health Promotion/ or exp *Health Care Policy/ or exp *Mental Health Programs/ or

exp *Program Evaluation/) and ((health$ or wellbeing$ or well-being$).ti. or

social-prescri$.ti,ab.) 19136

5 3 and 4 208

6 non-medical-refer$.ti,ab. 4

7 non-medical-care$.ti. 4

8 community-referr$.ti. 10

9 (referr$ and social-servic$).ti. 20

10 welzijn-op-recept$.ti. 1

11 1 or 2 or 5 or 6 or 7 or 8 or 9 or 10 314

12 limit 11 to (dutch or english) 293 🡪 Endnote WOR-1

13 (implem$ or facilitate$ or barrier$ or innov$ or evaluat$ or sustain$ or optimi$).ti,ab. 1009605

14 11 and 13 178

15 limit 14 to (dutch or english) 164 🡪 Endnote WOR-2

16 (standard$ or component$ or determinant$).ti,ab. 477177

17 14 and 16 28

18 limit 17 to (dutch or english) 25 🡪 Endnote WOR-3
