# Supplementary material for: Implement social prescribing successfully towards embedding: what works, for whom and in which context? A rapid realist review
Source: BMC Public Health. 2024 Jul 9;24:1836. doi: 10.1186/s12889-024-18688-3 (PMC11234751; doi:10.1186/s12889-024-18688-3)
Supplement: Supplementary file 2 — Supplementary Material 2 [file 12889_2024_18688_MOESM2_ESM.docx]

**Appendix 2 Guiding principles and summary of supporting enabling/hindering contexts, barriers, associated outcomes**

| **Guiding Principle 1:** **Create awareness of addressing wider determinants of health and the role social prescribing services can play.**   - Ensure there is some (formal) evidence about the effectiveness and improvement clients outcomes so health and care professionals as well as clients are more engaged. - Ensure that referrals to SP services are as normal as a referral to the medical sector to create a positive attitude to health and care professionals as well as clients. | | | | |
| --- | --- | --- | --- | --- |
| **Underlying CMO Configurations** | **Contextual factor** | **Mechanism** | **Outcome** | **Reference** |
| 1 | GPs refer patients to a linkworker/social domain for participation in an activity. These GPs want to know and needed to feel that patients were benefiting from social prescribing, because they were driven by a high quality care for patients | Getting regular feedback about how the patient was getting after their initial referral or get some formal evidence about the effectiveness and improvement patients outcomes makes that GPs may feel more enthusiastic about SP services | GPs were more likely to use SP services and were also more engaged | Aughterson & Baxter, et al (2020) |
| 2 | GPS are curious about formal evidence about the effective of SP services on patients outcomes | Too much focus on gathering formal evidence about effects of SP services on clients health outcomes makes less attention for the client’s needs, because they need to fill continuous questionnaires | Its possible research is getting in the way of the activities and support clients really need. | Aughterson & Baxter, et al (2020) |
| 3 | A good relationship between navigators and other partners (i.e. general practice staff and service providers) is important and promotes effective communication about clients | Feedback on services users journeys and outcomes to GPs and practice staff via navigators e.g. during regular meetings or a short periodic report | Helps GPs and other GP staff understand how patients progress after their referral and GPs can be more supported for patients | Penschany & Pappas, et al (2018) |
| 4 | SP services are relatively new and healthcare professionals may struggle to explain the SP services to patients | When healthcare professionals presented a more social and self-management solutions to the problems of clients , it may demotivate some patients as they had expected a medical solution. | This may result in some patients dropping out and not engaging further with Social Prescribing | Mofizul Islam, (2020) |
| 5 | Clients who are isolated for a long time and who are eligible for referring to social domain, they expecting that their needs are addressed in a medical way. These clients are also unfamiliar with SP types of services. | Clients who are prescribed a different approach than a medical one may get nervous about having to do something different from what they initially expected. | Clients can be reluctant to take up Social prescribing | Scott & Fidler, et al (2020) |
| 6 | Elderly, frequent flyer clients have a tendency to ring up the GP with general and broad complaints  Traditionally these complains would have been responded to medically | These issues are not being recognized that these complaints are more social in nature rather than medical | Means that GPs/SP services are starting to ask these frequent flyers differently with non-medical questions  Cultural change is created. | Scott & Fidler, et al (2020) |

| **Guiding principle 2: Ensure health and care professionals build trusting relationships with all involved stakeholders to create a cyclical referral process.**   - Create a shared understanding between all involved health and care professionals to feel confident working with which makes referrals more easily. - Ensure clients trust their health and care professionals so they share their problems and are more willing to participate in recommended community services. | | | | |
| --- | --- | --- | --- | --- |
| **Underlying CMO Configurations** | **Contextual factor** | **Mechanism** | **Outcome** | **Reference** |
| 1 | Newly implemented SP services that first focusses on creating buy in for SP services | For linkworkers it is important to build a trusting relationship with clients so they feel safe enough to share sensitivities about their personal lives | When linkworkers understand the personal circumstances of clients makes it possible to refer clients to an appropriate activities or services | Tierney & Wong, et al (2020) |
| 2 | Within GP practices, there are many part-time and locum GPs and with regularity high staff turnover there is no real preexisting relationship with clients due to high staff turnover | This lack of established relationships and knowledge about the clients hinders clients to trust their GP | The change in staff makes it difficult to spend enough time with clients to build a relationship | Aughterson, et al (2020) |
| 3 | When GPs are working at the practices for a long time and have face to face connections with the local communities | These trusting relationship between GPs and the local community, give GPs a sense of confidence to refer an clients to the local community. | That makes that clients can use SP services and can receive appropriate support | Aughterson, et al (2020) |
| 4 | Green health interventions as part of SP services, aim to integrate green health interventions in local services provision  Setting up new signposting and referral processes, either through setting up entirely new pathways or by integrating with existing pathways. | Each approach requites to build relationships and work together with health and care professionals to improve understanding and increase confidence about new activities or interventions for clients | It is then more likely to get new and more referrals to new activities or interventions | McHale & Pearsons, et al (2020) |
| 5 | There is limited awareness of SP services within the local communities | People in the local community find it important if the link worker comes from the same local community, so they can help build trust and understanding between SP services & local communities. | Helps engage communities with SP services and helps align services to communities needs | Khan & Ward, er al (2021) |
| 6 | Social Prescribing was no longer articulated as a linear referral pathway towards a predefined destination but more as a care network comprising different actors | Needs of clients can change over time and make that they moved back and forth across settings and sectors | It requires an ongoing and bidirectional coordination between care providers | (Calderon – Larranaga (2022). |
| 7 | The service was only intended as short-term signposting, most linkworkers and volunteers tried not to exceed 12-week support period stipulated in the service specification  C: some 12 week program is not long enough for people | linkworkers felt that some services-users needed longer tem support due to the complex nature of loneliness | Which should be result in being flexible in their delivery model to enable workers to tailor support to the services-users needs, and hightlight that some people need more than a short term signposting service. | Holding, et al (2020) |
| 8 | GPS found it challenging to persuade some patients to see a link worker or try a community activity | Clients can have a motivational threshold to surpass in order to agree to engage with social prescribing, or can have feelings like anxiety and depression which makes it difficult to try new activities or trying new things | have difficulties with joining new activities or trying new things | 127  Aughterson, et al (2020) |

| **Guiding principle 3: Invest in linkworkers’ skills and capacity so that they can act as a bridge between the sectors.**   - Ensure linkworkers are able to 1) connect different organisations, 2) create an overview of the local infrastructure, 3) provide support to (vulnerable) clients with (multiple) health needs. - Ensure linkworkers are supported enough in various ways to feel confident enough to fulfill these job. | | | | |
| --- | --- | --- | --- | --- |
| **Underlying CMO Configurations** | **Contextual factor** | **Mechanism** | **Outcome** | **Reference** |
| 1 | Patients with multiple and complex needs are referred through social prescribing to a link worker | Empathetic link workers with good knowledge of social support infrastructure available locally, gives client sense of agency and control over their time with non-imposing support | It haves beneficial impact on services users, but in particularly services users with multiple and complex needs | Bertotti & Frostick, et al (2018) |
| 2 | Social prescribing offers different levels of interventions. This ranges from straightforward signposting which requiring a detailed knowledge of local organizations available to a more intensive coaching-style intervention for patients who needed to overcome barriers before signposting. | Link workers who have different skills and have an educational background in psychotherapy, psychology, coaching and have experiences working in the voluntary sector as well as considerable listening and empathetic skills | Makes it possible to help patients better to a next step and create significant behavioral change. | Bertotti & Frostick, et al (2018) |
| 3 | SPs who are working in their role experienced emotional burden and feeling unable to help within the limits of their personal resources and at risk of burn-out | SPs who feel supported by a safe space to debrief their experiences, like one-to-one supervision and peer support as particularly valuable or interact with a team of social prescribers. | prevents them or a burnout and feeling isolated in their role and can also learn from other SPs how to keep professionals distance and being aware of setting boundaries | Rhodes, et al (2020) |
| 4 | SP services for acute MH issues (e.g. acute crisis like suicide)  Professionals are still searching for which clients are most suitable to SP services | Some professionals feel a sense of responsibility for taking on clients (e.g. those with acute MH needs) who would otherwise not be taken up by other/traditional services. Therefore, professionals want to be able to offer the client some services | There's no one size fits all approach to deciding which clients are appropriate or not, this requires a person-centred aprpoach | Scott, et al (2021) |
| 5 | Clients with a lot of mental health problems were referred to a linkworker  Linkworkers without a professional background are working with mental health issues | Link workers need more training to feel confident enough in the working they are doing with clients who have mental health issues | Can help clients with mental health problems appropriately and effectively  Protect themselves and the individuals they working with | Hazeldine, (2021) |
| 6 | SP services and addressing social needs is for primary clients relatively new to professional | Linkworkers need to build relationships with staff in order to be seen as credible and competent to primary care staff, so they trusted the linkworker with the client | Primary care staff get confidence in referring patient to a linkworker | Thierney, et al 2020 |

| **Guiding principle 4: Ensure clients receive appropriate support to improve their self-reliance and increase their community participation.**   - Ensure that clients receive appropriate support in resolving multi-problem issues to feel less dependent on health and care professionals. - Make sure clients feel adequately supported to participate in community and voluntary services activity. |
| --- |

| **Underlying CMO Configurations** | **Contextual factor** | **Mechanism** | **Outcome** | **Reference** |
| --- | --- | --- | --- | --- |
| 1 | The presence of a linkworker to remove feelings of discomfort and un familiarity from new situations | Feelings of clients about a past can be always there and persistent predominant feelings of uncertainty and discomfort. The presence of a linkworker is not always enough to enter unfamiliar fields of practices. | A other intervention is first needed to help this client, before the client can participate in a activity in a social domain. | Gibson, et al (2021) |
| 2 | A client was referred. | Linkworkers who contact patients directly after receiving a referral and give emotional and practical support to overcome barriers that often prevented them from engaging | Prevents dropouts and enable people to push themselves harder than they would have by themselves  Patient were more likely to participate | Calderon – larranaga. et al 2022 |
| 3 | SP services provided social group activities | When clients support each other in an informal manner, which created peer-support amongst clients | Clients self-reliance and social engagement is increased | Dayson, et al 2020 |
| 4 | The service was only intended as short-term signposting, most linkworkers and volunteers tried not to exceed 12-week support period stipulated in the service specification  C: some 12 week program is not long enough for people | linkworkers felt that some services-users needed longer tem support due to the complex nature of loneliness | Which should be result in being flexible in their delivery model to enable workers to tailor support to the services-users needs, and hightlight that some people need more than a short term signposting service. | Holding, et al (2020) |
| 5 | GPS found it challenging to persuade some patients to see a link worker or try a community activity | Clients can have a motivational threshold to surpass in order to agree to engage with social prescribing, or can have feelings like anxiety and depression which makes it difficult to try new activities or trying new things | have difficulties with joining new activities or trying new things | 127  Aughterson, et al (2020) |

| **Guiding principle 5 Invest in the aligning of structures, processes and resources between involved sectors to support the use of SP services.**   - Ensure there are shared resources and systems available between the sectors to collaborate and communicate across a diverse group of stakeholders. - Ensure there is structural funding so SP services can be offered permanently. | | | | |
| --- | --- | --- | --- | --- |
| 1 | A collaborative multi-sector approach with a diverse group op stakeholders is important to contributed towards a delayed implementation of delivery of SP | The lack of a targeted approach to strategic and robust project management to undertake all the coordination required for the program and the absence of a robust risk management system to be prepared for scenarios | This could be disrupt and delayed implementation and delivery of SP services | Penscheny & pappas, et al (2018) |
| 2 | GP acted as facilitate as facilitators for link workers | GPs offered an open environment for linkworkers and practical support by offering suitable location, access to the practices to speak with practice staff, access practice resources (wfi, patientinformation system, emai, printing and advertising in waiting room). Linkworkers feel welcome in the GP surgeries | It creates more collaboration between GP surgeries staff and linkworkers, more likely to connect to what clients need services contributes implementation | (Hazeldine, et al 2021) |
| 3 | Computerized system used as a directory & available services, but this directory is often out of date | The lack of an automated system to the social domain causes referral to traditional services because it feels time consuming | Reduced referrals to SP services and highlight potential need to redraw referral pathways to better include SP services | Scott, et al 2021 |
| 4 | SP services are not yet formalized as they are largely placed within VCSE sector and depend on volunteers to act as link workers | The lack of clear standards and boundaries for SP services makes that voluntary linkworkers are unequipped to do with some clients needs | Linkworkers needed to be train to help clients with complex needs  Has led to many concerns including for clients confidentiality  Has led to a call of clear accountability and governance structures for SP services | Islam, et al (2020) |
| 5 | Sustainability of SP interventions delivered across different organizations | Shared resources and systems are improved communication and built relationships between different organizations | Makes it easier to referring clients with mental health problems for community support  Clients have to wait longer and there is more change to disengage | Thomas, et al 2021 |
| 6 | Many SP organizations are in the voluntary sector and are isolated in from statutory services what content that information is not passed between organizations in a timely manner  There is no interoperability in IT systems | The lack of professional status of SP staff makes that there is no interoperability in IT systems | Leads to uncertainty about what kinds of information can or cannot share with them  Resulted in difficulties with systematic shared information | (Wood, et al 2021) |
